# Event and Comment

**Published:** 1914-11-15

**Authors:** 


// DENTAL REGISTER.

Vol. LXVIII. November 15, 1914. No. 11

### EVENT AND COMMENT.

THE FOUR YEAR DENTAL COURSE. The editor of the *Pacific Dental Gazette* in commenting on the proposal of the colleges in the Dental Faculties Association of American Universities to extend the time required for completing the dental curriculum to four years, makes a statement that if these schools would fully occupy the time now at their disposal they would teach the same number of hours as the proposed extended course will include. The editor of the *Gazette* figures that there are approximately 4,200 teaching hours in the present eight months course, if all the time was properly occupied, and that the schedule submitted by the chairman of the committee outlining a tentative schedule for the four year course does not suggest a greater number of hours. There are several misconceptions in these premises which will materially affect the conclusions. We do not care to make a complete or critical analysis, but we would like to say that in the present nine months term schools there are, clear of all holidays, 200 teaching days, counting four hours on Saturday as a full day, and the number of hours of class and laboratory exercises will average 8 hours per day for five days and four hours on Saturdays, making 44 hours per week. There are 34 weeks of actual teaching not counting the opening week, nor the last week which is commencement week. We will thus see that there are actually 1,496 teaching hours instead of 696 as figured by the editor of the *Gazette.* It may be true that in some schools so many hours are not scheduled and in some schools more hours are actually scheduled. It is also true undoubtedly that some students put in more time than is actually scheduled for them. Now if our figuring is correct and we happen to know that it is substantially correct we are at present teaching 4,500 hours in the three year course instead of 4,200, and should a fourth year be added we should be teaching 6,000 hours. So much for figures.

This, however, is not the objective sought in the extended course. The editor of the *Gazette* pleads for better teaching, and a more careful study of the individual student that his peculiar characteristics of mind and manipulation talents may be cultivated to better purpose in the time at present at our disposal. We are wondering if our critic has ever conducted a dental college and had to employ teachers and find the money to pay them salaries that would justify them in devoting their lives to teaching exclusively. The suggestion is made that teachers should be employed only after having passed rigid examinations to determine their knowledge and capacity for imparting it to students. All of this reads good, but just stop to think what it would cost to engage a faculty that could qualify on such a basis. There isn't a dental college teacher, so far as we know, that is receiving a salary of over $5,000 per annum for full time services, and not one such man is paid what he could earn as a practitioner. Now if we were to equip our dental colleges with sufficient teachers of this grade to do the work on the plan suggested it would cost at least $50,000 to pay the faculty alone to say nothing of other necessary expenses, to care for schools having the smallest attendance. Moreover, under present conditions it is not practicable to individualize dental instruction, its cost would be prohibitive. At present the dental student thinks his college fee of $150 per annum is exorbitant, and many of our professional writers on business topics think an expense of $2,000 for a dental education is out of proportion to the income the average dental graduate earns the first five years he is in practice. It seems for the present, at least, that dental education must be made as inexpensive as it is practicable to make it and secure tolerable preparation until the supply of graduates is materially increased and this can only be done by imparting instruction to students in classes rather than as individuals or even in small groups. It is a great gain for a school to have in its faculty even one broad minded and skillful instructor, and we believe most of our schools have more than one such, but for economic reasons it is necessary that much of the routine instruction shall be done by less expensive and possible less skillful instructors. Of course we do not advocate here the delegation of so important a function as teaching to the tyro, but we simply mean to say that it is practically impossible to man entirely our schools with ideal teachers. Such a possibility would certainly be a great gain, and we should just love to be able to realize it even in a dream.

This, however, is not all we had in mind to say when we took up our pen to comment on this editorial. The teachers in our dental colleges of long experience, who have carefully considered the matter of education for dentistry, will undoubtedly agree with us in the assertion that the reason dental students do not make a better showing in their theoretical studies before State Examining Boards is that they do not have sufficient time during their college course to do the necessary reading to make them fully intelligent thinkers about the work presented each day to them in their classes. It is a notorious fact that students buy only such text books as are absolutely required and where a quiz compend can be substituted for a text it is the practice to buy such unsatisfactory books and depend upon them, and such notes as the student can make of the lectures, for his knowledge. In fact, the time a student has outside of the class room and laboratories for study is so limited at best that he is driven to take advantage of every short cut to knowledge that may be available. Now every one can see that however skillful the instructor might be if his students only memorize facts, he can never hope to make thinkers or scholars of them. If the average dental practitioner of today were to attend a dental convention and have as many new things to think about presented to him as the average dental college give to its classes every day he would be tired for a week. To our mind, then, the reason for a longer course of study is not that more subjects necessarily may be added to the curriculum, but that the subjects now taught may be more thoroughly impressed and that time may be given for fuller comprehension and assimilation by the student. The student should have a fair chance to arrange his knowledge and perhaps to correlate his scientific and artistic instruction and to translate both into terms of his own understanding. When we stop to think about it, it is remarkable that a young man just from high school, where he is taught to recite so many pages from a text book, takes up college work and tries to do a full days work every day succeeds at all, when the method of instruction is so different. It would seem to us that the first year of college work should be so planned as to prepare the school boy to assimilate the new method of instructions. which as now given pre-supposes the ability of the school boy to do his own thinking. Our teaching in colleges is largely done by men who are specialists in their own lines and they are not expected to incorporate allied subjects in their lectures. The time was when knowledge was scarce and teachers and students were few, then there were intimate relations which gave opportunity to develop the students thinking capacity, and to satisfy his desires because they could be known. President Garfield once said that his idea of a college education was to be seated on a bench opposite Mark Hopkins, the illustrious president of Williams College.

Some such ideal evidently was in the mind of the editor of the *Pacific Gazette* when he wrote his criticism of the proposed college course. If we could have such ideal conditions everywhere, the large schools would have to go, and there would be no standard of education for all to strive after, and we should develop many men of genius and character perhaps, but it would be expensive and we doubt if the needs of the people would be better cared for than under present conditions. It looks to us as though our system of education must stand, and that our plain duty is to do all that is possible or practicable to improve it by putting our wits to work on plans that will make it more effective in producing better prepared graduates. We are inclined to believe that more time, properly proportioned and better utilized will bring this much desired progress in dental education. It is a subject that will stand further discussion.

A SENSIBLE PROGRAMME. The Indiana State Dental Society will this year try the experiment of having a program contributed entirely by its own members. To our notion this will be the most interesting experiment of the year. There is plenty of talent in every state to provide papers for discussion and sufficient clinics to interest and instruct all who will give their attention to them. It is not always the well considered thought in the imported paper that brings out the profitable discussion, too often the subject is so technical or scientific that no one feels competent to either criticise or amplify its material and no enthusiasm and little impression is made. A simple home-made paper, out of one's experience, will many times create a lot of discussion and interest. A great benefit we imagine will result from the induction of more of the modest yet capable thinkers and doers into the work of providing the program. No one takes quite the same interest in the big dinner as the cook herself. No one enjoys the dental convention quite so much as the people who have taken some part in its doings. We trust the Indiana programme will be filled with "never before heard of's." We should be glad to attend that meeting. It will develop more originality than wisdom perhaps, but it will be none the less valuable on that account. It may not meet the approval of every one the first year but it should be kept up long enough to introduce some new people to the reading world.

